# Controlled-rate freezer cryopreservation of highly concentrated peripheral blood mononuclear cells results in higher cell yields and superior autologous T-cell stimulation for dendritic cell-based immunotherapy

**DOI:** 10.1007/s00262-012-1262-0

**Published:** 2012-04-22

**Authors:** Timo Buhl, Tobias J. Legler, Albert Rosenberger, Anke Schardt, Michael P. Schön, Holger A. Haenssle

**Affiliations:** 1 Department of Dermatology, Venereology and Allergology, University Medical Center Göttingen, Robert-Koch-Str. 40, 37075 Göttingen, Germany; 2 Department of Transfusion Medicine, University Medical Center Göttingen, Göttingen, Germany; 3 Genetic Epidemiology, University Medical Center Göttingen, Göttingen, Germany

**Keywords:** DC, Dendritic cells, Cryopreservation, Cellular immunotherapy, Cell yields

## Abstract

Availability of large quantities of functionally effective dendritic cells (DC) represents one of the major challenges for immunotherapeutic trials against infectious or malignant diseases. Low numbers or insufficient T-cell activation of DC may result in premature termination of treatment and unsatisfying immune responses in clinical trials. Based on the notion that cryopreservation of monocytes is superior to cryopreservation of immature or mature DC in terms of resulting DC quantity and immuno-stimulatory capacity, we aimed to establish an optimized protocol for the cryopreservation of highly concentrated peripheral blood mononuclear cells (PBMC) for DC-based immunotherapy. Cryopreserved cell preparations were analyzed regarding quantitative recovery, viability, phenotype, and functional properties. In contrast to standard isopropyl alcohol (IPA) freezing, PBMC cryopreservation in an automated controlled-rate freezer (CRF) with subsequent thawing and differentiation resulted in significantly higher cell yields of immature and mature DC. Immature DC yields and total protein content after using CRF were comparable with results obtained with freshly prepared PBMC and exceeded results of standard IPA freezing by approximately 50 %. While differentiation markers, allogeneic T-cell stimulation, viability, and cytokine profiles were similar to DC from standard freezing procedures, DC generated from CRF-cryopreserved PBMC induced a significantly higher antigen-specific IFN-γ release from autologous effector T cells. In summary, automated controlled-rate freezing of highly concentrated PBMC represents an improved method for increasing DC yields and autologous T-cell stimulation.

## Introduction

Based on their unique abilities to initiate, tolerate, or abrogate immune responses, dendritic cells (DC) have become major candidates for immunotherapy. Especially, the capacity of DC to take up and present antigenic epitopes on major histocompatibility complex (MHC)-I and MHC-II to CD4+ and/or CD8+ T cells account for their promising potential in clinical trials [1, 2]. Most research has focused on DC-based cancer immunotherapy [3, 4], which is spearheaded by the FDA approval of sipuleucel-T (APC8015), a cellular vaccine for patients with metastatic prostate cancer based on enriched autologous antigen-presenting cells briefly cultured with a fusion protein of prostatic acid phosphatase and GM-CSF [5]. Other areas of intense research for DC-based or DC-targeted immunotherapy include infectious diseases, allergy, autoimmunity, and transplantation medicine [6–8].

Ex vivo manipulation of DC for reinfusion into patients as well as monitoring of cellular function during immunotherapeutic clinical trials usually require cryopreservation of cells. Over the last several years, cryopreservation protocols for fully differentiated DC after a broad variety of maturation agents, antigen loading, and costimulatory strategies were validated [9–11]. Recently, an in-depth analysis of cryopreservation of different DC and progenitor cells revealed superiority of cryopreserved monocytes for DC generation over all other types of cryopreserved immature, semi-mature, and mature DC regarding their cell viability, surface markers, and functional properties [12]. DC cryopreservation is usually performed by standard cryopreservation containers where cell tubes are surrounded by isopropyl alcohol (IPA) and simply placed inside a −80 °C freezer.

Generally, cell damage in freeze–thaw processes is either caused by extensive cell dehydration (“solution effect”), by intracellular ice crystallization (“mechanical damage”), or a combination of both. Cell dehydration commonly occurs during low-rate freezing, while rapid freezing usually results in ice crystallization [13, 14]. Cryopreservation by controlled-rate freezers (CRF) is thought to minimize both effects through continuous adjustment of the temperature decline according to the actual temperature of the cryo specimen thus compensating for fusion heat and minimizing supercooling effects [15–17]. Although CRF are routinely used for cryopreservation of autologous peripheral blood progenitor cells (PBPC) for transplantation [18], this approach has not been applied for clinical studies with DC immunotherapy. To the best of our knowledge, a head-to-head comparison of phenotypic and functional properties of PBMC for DC immunotherapy after uncontrolled- and controlled-rate freezing procedures has not been reported.

## Materials and methods

### PBMC preparation and cryopreservation

PBMC from IPA and CRF cryopreservation are termed “IPA-PBMC” and “CRF-PBMC” throughout this manuscript. DC generated from non-cryopreserved (NC) PBMC is referred to as “NC-DC”, and DC generated from IPA- and CRF-cryopreserved PBMC as “IPA-DC” and “CRF-DC”, respectively.

**Fig. 1:**
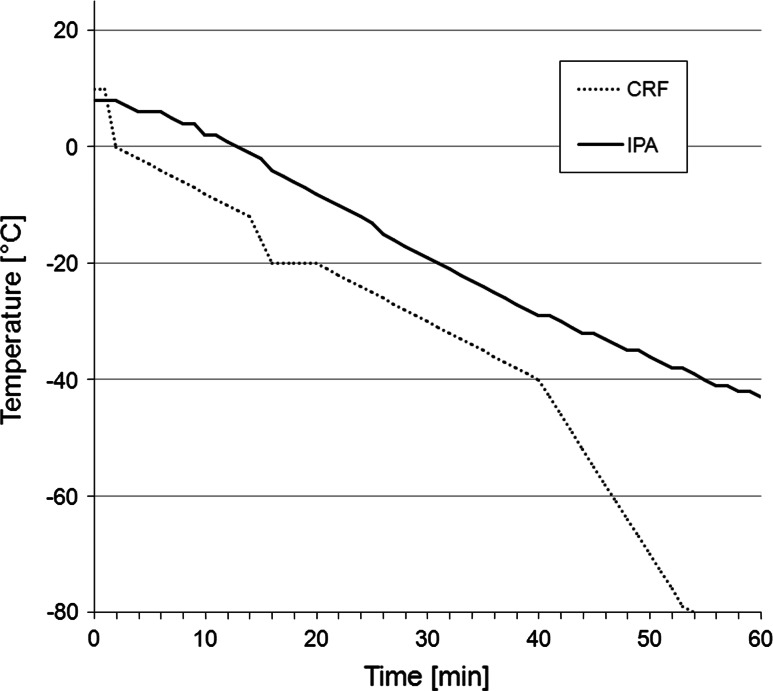
Temperature profile of controlled-rate freezer (CRF) and isopropyl alcohol (IPA) containing cryopreservation containers. The CRF cryopreservation procedure for 2 × 10^8^ PBMC/ml was configured by the following program parameters: −10 °C/min to 0 °C, −1 °C/min to −12 °C, −4 °C/min to −20 °C, hold for 5 min at −20 °C (preventing the crystallization heat that may result in a slight temperature increase at this stage), −1 °C/min to −40 °C, −3 °C/min to −80 °C, hold for 6 min at −80 °C. IPA freezing reached a temperature of −42 °C after 60 min. The rate of temperature decline in the IPA containers constantly decreased, reaching −80 °C finally after 300 min

Leukapheresis products of healthy donors were obtained by a COBE Spectra™ cell separator (Gambro BCT Inc., Lakewood, CA, USA) using the MNC settings as previously described [19]. The study was approved by the local ethics committee, and written informed consent was obtained from all donors. Preparation of PBMC was performed by density gradient centrifugation, aliquots of isolated PBMC were used for immediate cell culture and for cryopreservation in freezing medium consisting of 20 % dimethyl sulfoxide (DMSO; Sigma-Aldrich, St. Louis, MO, USA), 40 % fetal calf serum (FCS; PAA, Pasching, Austria), and 40 % RPMI 1640 (PAA, Pasching, Austria). PBMC for cryopreservation were immediately transferred into 1-ml cryopreservation tubes (Greiner bio-one, Frickenhausen, Germany) at a concentration of 2 × 10^8^ in 1 ml of freezing medium and either frozen at a cooling rate of approximately −1 °C/min to −80 °C by using a standard freezing container with isopropyl alcohol (Nalgene, Roskilde, Denmark) or were frozen by a computer-assisted controlled-rate freezer (Planer Kryo10 SerieII, Messer Griesheim, Krefeld, Germany) with a temperature-controlled program to −80 °C (details on programming of CRF in Fig. 1). After freezing procedures, samples were stored in a liquid nitrogen container. For further analysis, frozen cells were rapidly thawed in a water bath at 37 °C until detachment of cells became visible, and then washed in RPMI 1640.

### DC generation and maturation

Immature DC (iDC) were generated from a plastic-adherent cell fraction of PBMC. Adherent cells were grown in RPMI 1640 supplemented with 10 % FCS, 100 IU/ml penicillin, 100 μg/ml streptomycin (PAA, Pasching, Austria), 800 IU/ml GM-CSF, and 1,000 IU/ml IL-4 (Immunotools, Frisoythe, Germany) and cultured for 6 days as described earlier [20]. For maturation of iDC, day 6 cultures were supplemented with 18 μg/ml of poly(I/C) (Sigma-Aldrich, St. Louis, Missouri, USA) for 48 h. As control, iDC were cultured for another 2 days in RPMI 1640 containing 10 % FCS, 800 IU/ml GM-CSF, and 1,000 IU/ml IL-4. On day 8, all supernatants were collected and stored at −20 °C for cytokine profile analysis.

### Flow cytometry

Antigen expression was determined using antibodies against the following antigens: CD3, CD4, CD8, CD14, CD19, CD83, CD86, HLA-DR, or the respective isotype-matched controls (all Invitrogen, Carlsbad, CS, USA except CD83-Ab from Beckman Coulter, Krefeld, Germany). Integrity of the plasma membrane and thereby cell viability was assessed by propidium iodide staining (Sigma-Aldrich, St. Louis, MO, USA). Cells were washed and immediately analyzed by flow cytometry (FACSCanto II with DIVA software, BD Biosciences, San Jose, CA, USA). Dead cells and debris were gated out on the basis of their light scatter properties.

### Total protein quantification

During DC generation, the adherent cells were harvested on day 1, day 4, and day 6 by repeated scraping to collect all cell material for protein quantification (since whole cell harvesting was not possible due to adhesion). Pellets were stored at −20 °C until total protein quantification by Bradford protein assay (Bio-Rad Laboratories, Munich, Germany).

### Cytokine profiling in cell culture supernatants

Cells were plated on day 6 at a density of 2 × 10^6^ iDC per well in 6-well plates in RPMI 1640 containing 10 % FCS, 800 IU/ml GM-CSF, and 1,000 IU/ml IL-4. After maturation by poly(I/C) for 48 h, cell culture supernatants were collected and stored at −20 °C for cytokine profile analysis. Nitrocellulose membranes spotted with capture antibodies against 36 human cytokines (R&D, Minneapolis, MN, USA) were used for cytokine profiling of cell culture supernatants. The assay was performed according to the manufacturer’s instructions. After adding a chemiluminescent agent (ECL Plus Detection Reagents, GE Healthcare, Buckinghamshire, UK), blots were recorded by image reader LAS-4000 (Fujifilm Europe, Düsseldorf, Germany) and submitted to an automated digital analysis using Fujifilm Multi Gauge software (Fujifilm Europe, Düsseldorf, Germany).

For verification of results concerning the IL-12p70 (bioactive heterodimer) concentration in supernatants, quantitative analysis was performed by ELISA assay (R&D Systems, Minneapolis, MN, USA) according to the manufacturer’s instructions.

### Proliferative response of allogeneic CFDA-SE-labeled T cells

In order to measure proliferative responses of allogeneic T cells to cryopreserved or freshly prepared DC, respectively, T cells were separated from PBMC by magnetic cell sorting (MACS) using CD3-MicroBeads (Mitenyi Biotec, Bergisch Gladbach, Germany). FACS analysis revealed a purity of >95 %. For carboxyfluorescein diacetate *N*-succinimidyl ester (CFDA-SE) proliferation assays, CD3+ T cells were stained with CFDA-SE (Invitrogen, Oregon, USA) according to the manufacturer’s instructions. Day 8 DC were cultured with allogeneic CFDA-SE-labeled CD3+ T cells in triplicates in flat-bottom 96-well plates in a final volume of 200 μl/well RPMI 1640 with 10 % FCS. 2 × 10^5^ T cells were stimulated with 2 × 10^4^ DC (ratio 10:1) [20]. After 5 days, cocultures were washed and assessed for CFSE staining of T cells by flow cytometry.

### Enzyme-linked-immunospot (ELISPOT) assay of DC with autologous PBMC

During DC maturation between day 6 and 8, DC cell cultures were supplemented with 50 μl/ml tetanustoxoid (TTX, a kind gift of Novartis Behring, Marburg, Germany) as recall antigen. On day 8, TTX-loaded DC resulting from the different cryopreservation protocols were washed separately and cultured in a 24-well plate with freshly thawed autologous PBMC (all cryopreserved by IPA protocol) for 7 days for expansion of antigen-specific memory T cells [2 × 10^6^ PBMC cocultured with 2 × 10^5^ DC (ratio 10:1), or 6.6 × 10^4^ DC (ratio 30:1)]. For improving further expansion and survival of T cells, 12 U/ml IL-2 (R&D Systems, Minneapolis, MN, USA) were added on day 10. After 7 days of coculture, cells were washed and analyzed by ELISPOT assays in order to quantitate TTX-driven IFN-γ release of effector T cells.

For IFN-γ ELISPOT, nitrocellulose-bottomed 96-well plates were pre-coated with capturing anti-IFN-γ mAb (Mabtech, Nacka Strand, Sweden). Briefly, 5 × 10^4^ non-adherent cells of re-stimulated cultures were incubated in triplicates with 5 × 10^4^ TTX-loaded autologous PBMC in 200 μl ELISPOT medium (RPMI 1640, 10 % FCS, 100 U/ml penicillin, 100 μg/ml streptomycin) for 48 h at 37 °C and 5 % CO_2_. Spots were stained using a Vectastain^®^ Elite kit (Vector Laboratories, Burlingame, CA, USA) and counted by a computer-assisted video imaging analysis system (AID EliSpot Reader System, Autoimmun Diagnostika, Strassberg, Germany). After configuration of software, all ELISPOT plates were analyzed in one single pass. No spots were added or removed manually.

### Statistical analysis

All figures show mean ± standard error of observed values or mean and observed values. The *F* tests of the ANOVA model were performed using the option ANOVAF, which is similar to the method described by Brunner et al. [21]. The considered outcome measures were regarded as logarithmized to achieve a better model fit. Normality was checked visually (via histogram and q–q-plot of the residuals) and by performance of a Shapiro–Wilk test. The level of significance was set to α = 0.05. If the closed test principle could not be applied, we corrected for multiple testing by Tukey–Kramer. Additionally, we performed a nonparametric Kruskal–Wallis test to ensure homogeneity in individual (directly observed) divergence of method-related specific INF-γ spots between the ratios 1:10 and 1:30 of the ELISPOT experiments. All analyses were performed using SAS 9.2 software (SAS Institute Inc., Cary, NC, USA).

## Results

### Cell yield of immature DC depends on method of PBMC cryopreservation

**Fig. 2:**
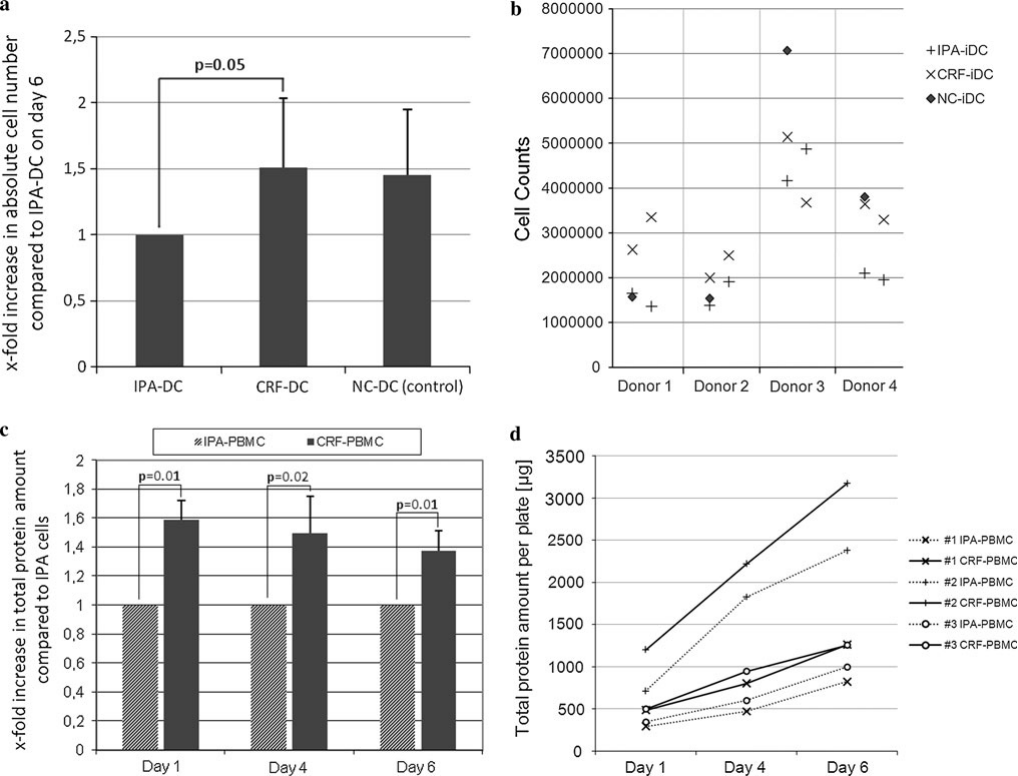
**a** Cell counts of immature DC on day 6, using a Neubauer counting chamber. The observer was blinded to the cryopreservation protocol used. Cell numbers are shown in relation to IPA-DC (set to 1.0 for each individual experiment). **b** Underlying original data of (**a**) as scatter graph: Four individual donors were analyzed (*n* = 4 for NC-DC). Two independent experiments of each donor’s IPA- and CRF-cryopreserved PBMC were performed (*n* = 8 for IPA-DC and *n* = 8 for CRF-DC). **c** Total protein per plate on day 1, 4, and 6 of cell culture after cryopreservation by IPA or CRF (Bradford protein assay, *n* = 3 different donors). CRF-PBMC protein amounts are shown in relation to IPA-PBMC protein amounts (set to 1.0 for each individual experiment). **d** Underlying original data of (**c**) shows total protein amounts per plate (μg) on day 1, 4, and 6. IPA-cells are depicted using a *dotted line* whereas CRF-cells are shown with *continuous lines*. Each of the markers “+, x, and o” indicate results of the same donor (*n* = 3 different donors)

For generation of iDC, plastic-adherent PBMC, freshly prepared or recovered from cryopreservation, were differentiated into non-adherent iDC by adding GM-CSF and IL-4 to cultures for 6 days. Cell yields of CRF-iDC on day 6 almost equaled cell numbers of freshly prepared iDC (observer blinded to protocols). Cell numbers of CRF-iDC exceeded IPA-iDC by a factor of 1.51 ± 0.53 (*p* = 0.05, Fig. 2a, b). A considerable donor-dependent variability of overall DC yields was noted. Therefore, the resulting cell numbers after experiments with NC-PBMC, IPA-PBMC, and CRF-PBMC were compared intra-individually. Identical cell numbers were pre-plated on day 0 in all experiments. Due to the plastic-adherence of DC progenitors, harvesting without disrupting the cell integrity was only possible at day 6 or later.

To reassess the results of day 6 cell counts, additional whole protein analysis of culture plate contents after repeated thorough scraping and microscopically ensured clearance of all cells was performed on days 1, 4, and 6. Whole protein quantification showed significantly higher protein amounts per culture plate for CRF-PBMC in comparison with IPA-PBMC and thus confirmed results of cell counts (Fig. 2c, d). Of note, an increased protein amount of CRF-PBMC was already observable after 1 day of culture. Flow cytometry analysis of NC-iDC, IPA-iDC, and CRF-iDC on day 6 revealed no differences in cell size or granularity (data not shown), underlining that the additional protein amount of CRF-iDC resulted from higher cell numbers.

### CRF-iDC and non-cryopreserved iDC show comparable phenotypic properties and maturation markers

**Fig. 3:**
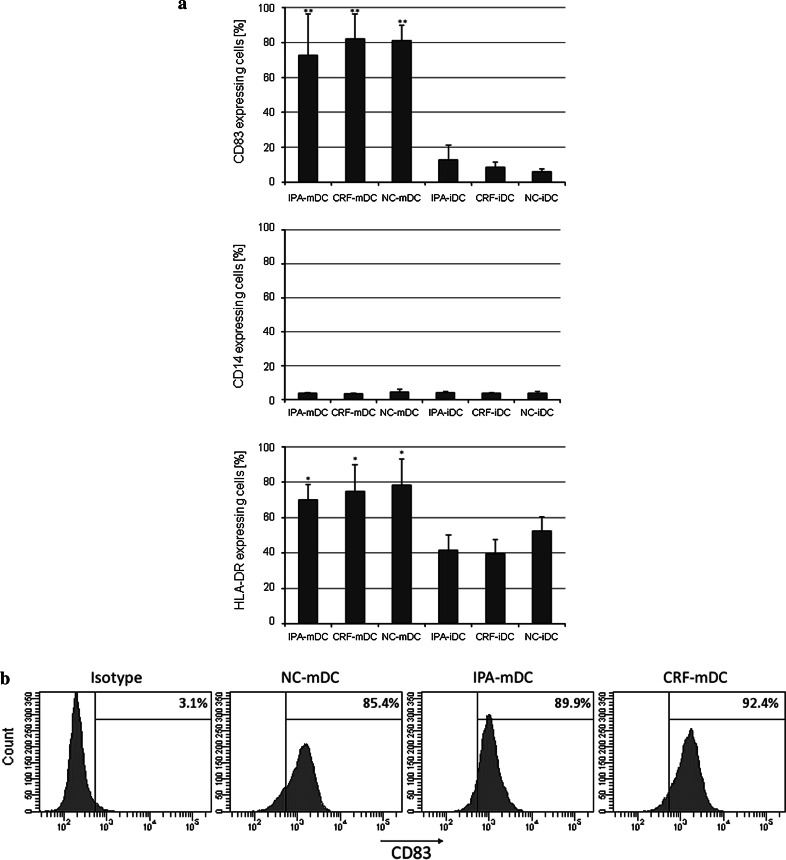
**a** Fraction of cells (%) expressing CD83, CD14 and HLA-DR by FACS analysis on day 8. After differentiation of day 6 iDC, cells were either kept in cultures under constant conditions for another 48 h (IPA-iDC, CRF-iDC, NC-iDC) or were supplemented with poly(I/C) for 48 h (IPA-mDC, CRF-mDC, NC-mDC). *n* ≥ 3 for NC-DC (controls). Significant differences were observed between iDC and mDC regarding CD83 (** *p* < 0.01) and HLA-DR-expression (* *p* = 0.01). No significant differences of CD14 expression in relation to the method of cryopreservation were noted (*p* = 0.35). **b** Histograms for CD83 staining of mature DC [positive fraction (%)], prepared from NC-PBMC, IPA-PBMC, and CRF-PBMC. One representative experiment of the series depicted in (**a**) is shown. Furthermore, mean fluorescence intensity of CD83 did not show significant differences between cryopreservation protocols

Day 6 NC-iDC, IPA-iDC, and CRF-iDC showed loss of CD14-expression to 2.7 ± 0.8 %, 2.9 ± 2.3 %, and 2.8 ± 1.2 % (mean ± standard deviation), respectively. The maturation marker CD83 remained low with 5.2 ± 3.7 % for NC-iDC, 2.7 ± 3.7 % for IPA-iDC, and 5.2 ± 4.2 % for CRF-iDC (data not shown). Maturation was induced by addition of the Toll-like-receptor (TLR) 3 activator poly(I/C) for 48 h, controls were kept under identical culture conditions without poly(I/C) addition. Staining of day 8 iDC (controls) and mature DC (mDC) for the representative surface markers CD83, CD14, HLA-DR again showed no significant differences between freshly prepared and cryopreserved mDC (Fig. 3). Noteworthy, CRF-mDC showed an identical strong upregulation of the maturation marker CD83 (82.0 ± 14.1 %) as freshly prepared DC (81.1 ± 8.7 %), while CD14-expression remained low (<5 %) after all three protocols. As expected, supplementation of cultures with poly(I/C) resulted in a statistically significant increase in CD83− (*p* < 0.01) and HLA-DR-expression (*p* = 0.01).

### Cytokine profiles of CRF-mDC, IPA-mDC and freshly prepared mDC

**Fig. 4:**
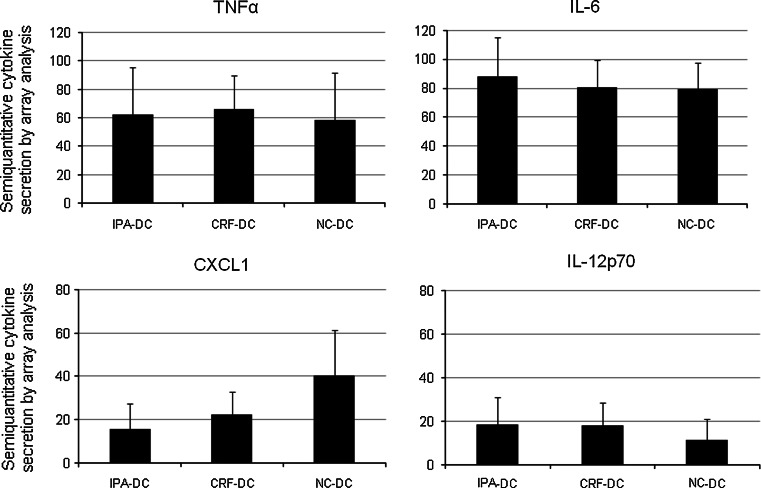
Selected cytokine profiles in cell culture supernatants obtained by cytokine arrays (*n* = 4 different donors). Of all 36 analyzed cytokines, only the secreted CXCL1 and IL-12p70 amount (*lower row*) differed between fresh DC and DC after cryopreservation protocols. IL-6 and TNFα (*upper row*) are depicted representatively (production of C5a, CCL1, CCL2, CCL5, CD54, CXCL10, CXCL11, IL-1RA, IL-8, IL-16, MIF, MIP-1α, MIP-1β, and Serpin E1 not shown)

Cell culture supernatants were collected after the day 6–8 maturation process for analysis of cytokine secretion by nitrocellulose membranes spotted with antibodies directed to 36 cytokines. Semi-quantitative analysis of the spot sizes of 32 cytokines revealed no differences between freshly prepared mDC, IPA-mDC, and CRF-mDC. In contrast, expression levels of CXCL1 and IL12p70 repeatedly differed between preservation protocols. While CXCL1 secretion levels of NC-mDC were twofold higher as compared to IPA-mDC, IL12p70 levels of NC-mDC were reduced by 50 % in relation to CRF-mDC (Fig. 4). The differences of IL12p70 levels were confirmed by ELISA analysis, revealing almost identical mean results, albeit the difference did not reach statistical significance (IPA to NC: *p* = 0.07, CRF to NC: *p* = 0.07; data not shown). No significant changes in cytokine array analysis were observed for C5a, CCL1, CCL2, CCL5, CD54, CXCL10, CXCL11, IL-1RA, IL-8, IL-16, MIF, MIP-1α, MIP-1β, Serpin E1, IL-6, and TNFα (Fig. 4 shows representative results of the latter two). At or below the detection limit were the amounts of CD40L, G-CSF, IFNγ, IL-1α, IL-1β, IL-2, IL-5, IL-10, IL-13, IL-17, IL-17E, IL-23, IL-27, IL-32α, CXCL12, and sTREM-1.

Expression of GM-CSF and IL-4 was not evaluated due to external supplementation with these cytokines during cell culture. For the majority of the 36 cytokines an inter-donor variability was noted.

### CRF-mDC demonstrate identical allogeneic, but superior autologous T-cell stimulatory capacity compared with IPA-mDC

**Fig. 5:**
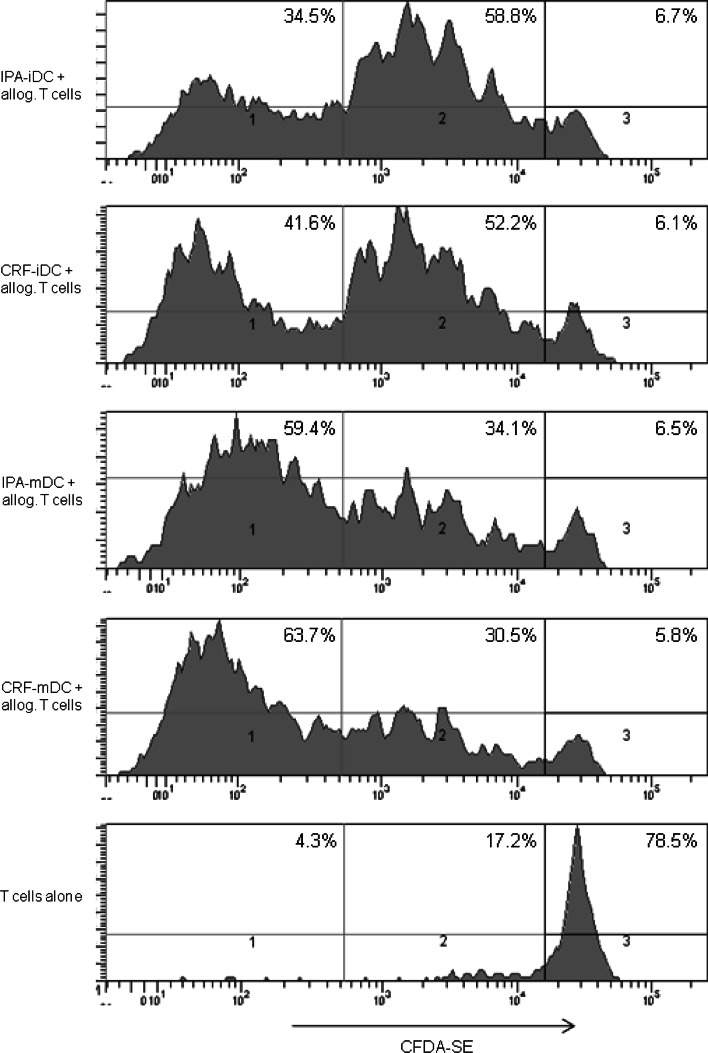
For comparison of T-cell stimulatory capacity of mDC derived from the different cryopreserved PBMC, proliferation assays with allogenic T cells were performed. T cells were separated from allogenic PBMC by MACS, labeled with CFDA-SE, and then cultured with mature DC (2 × 10^5^ T cells stimulated with 2 × 10^4^ DC, ratio 10:1) in triplicates for 5 days. After harvesting of the cells, CFDA-SE staining was assessed by flow cytometry. The figure shows one representative experiment out of three performed (*n* = 3 different donors). We defined three levels of T-cell proliferation activity: (1) high proliferation (>5 cell divisions), (2) low proliferation (1–5 cell divisions), (3) no proliferation

To evaluate functional DC properties after the different cryopreservation protocols, day 8 mDC were cultured with allogeneic CFDA-SE-labeled T cells for 5 days in a head-to-head comparison. CFDA-SE staining was analyzed by flow cytometry to determine proliferation of T cells. No significant differences in allogeneic T-cell proliferation between the different cryopreservation protocols or freshly prepared DC were found. Proliferation rates showed a strong donor dependency, supposedly because of the individual extent of MHC mismatch between DC and T cells. As expected, T cells cocultured with iDC of both cryopreservation protocols showed a low level of proliferation (1–5 cell divisions), whereas the majority of T cells cocultured with mDC reproducibly divided more than 5 times (Fig. 5).

**Fig. 6:**
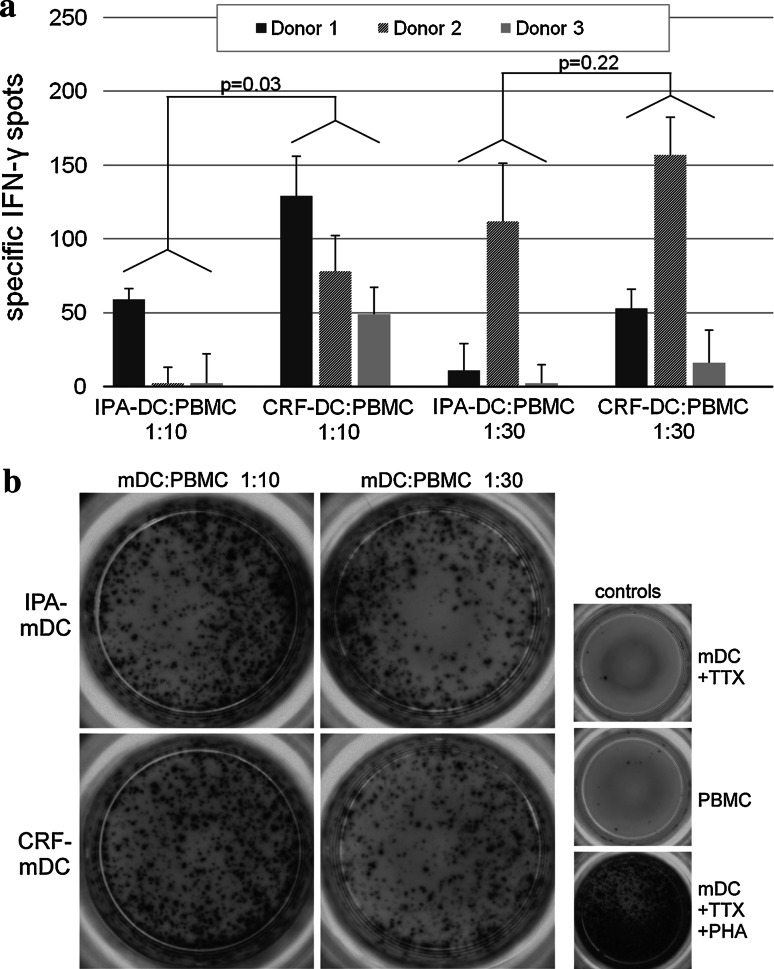
**a** Antigen-specific IFN-γ release of autologous PBMC after coculture with different tetanus toxoid- (TTX) loaded mDC (*n* = 3 different donors). Before determination of IFN-γ release in ELISPOT assay, TTX-loaded mDC were cultured with autologous PBMC for 1 week to allow expansion of antigen-specific T cells. Two different ratios were used as follows: 2 × 10^6^ PBMC+ 2 × 10^5^ mDC (ratio 10:1) and 2 × 10^6^ PBMC+ 6.6 × 10^4^ mDC (ratio 30:1). For IFN-γ ELISPOT, 5 × 10^4^ non-adherent cells after two rounds of stimulation were incubated in triplicates with 5 × 10^4^ TTX-loaded autologous PBMC for 48 h. Analysis of spots was performed by a computer-assisted video imaging analysis system. ELISPOT plates were analyzed in one single pass, and no spots were added or removed manually. TTX-specific spots were calculated by subtracting spots induced by unloaded control mDC from spot numbers induced by TTX-loaded mDC. **b** Images of the different cell subsets of donor 1 in IFN-γ ELISPOTS used for computer-assisted spot analysis. All experiments were performed in triplicates

In addition, we evaluated DC after different cryopreservation protocols for their autologous T-cell stimulatory capacity against the recall antigen tetanus toxoid (TTX) by coculturing TTX-loaded mDC with autologous PBMC at ratios 1:10 and 1:30 (DC/PBMC) for 7 days. Resulting cells were phenotypically analyzed by flow cytometry, counted, and transferred to a 96-well plate as responder cells for IFN-γ-ELISPOT assay at identical cell numbers. Freshly thawed autologous PBMC were used as stimulator cells. ELISPOT assays with CRF-DC from three different donors revealed TTX-specific IFN-γ-spots persistently, while three of the six IPA-DC batches failed to induce a specific response (Fig. 6a, no specific response seen in IPA-DC of donor 2 at 1:10 ratio, and donor 3 at 1:10 and 1:30 ratio.). In two of the three donors, a DC/PBMC ratio of 1:10 during the expansion phase yielded the highest number of specific spots. Donor 2 revealed a higher specific spot number at ratio 1:30 compared with 1:10 DC/PBMC ratio. Since we did not perform titration experiments for best stimulation rates in each donor, we could not predict which ratio might be optimal in every individual donor. We observed a significant overall effect associated with the method of cryopreservation: Within a DC/PBMC ratio of 1:10, we observed 21.4 more specific IFN-γ spots by CRF compared with IPA (*p* = 0.03). Although the sample size is small, comparison of the IFN-γ spots of the same donor showed the highest increase in specific spots (65.7 ± 15.0) for CRF-DC compared to IPA-DC at ratio 1:10.

## Discussion

We present data in favor of using highly concentrated PBMC, cryopreserved by a computer-assisted controlled-rate freezer (CRF), to reach a maximum yield of viable iDC after 6 days of culture. In our experiments, uncontrolled freezing by using standard isopropyl alcohol (IPA) resulted in significant cell loss during differentiation into iDC. Increased cell recovery after cryopreservation is a crucial aspect during clinical application of DC immunotherapy, especially with respect to human and financial resources and to prevent additional leukapheresis for the patient. It is thought that only a small fraction of injected DC finally reaches the lymph nodes depending on the injection route (e.g., ≤4 % after intradermal injection [22]). Moreover, a recent meta-analysis revealed a positive influence of higher DC doses on the clinical response rate in DC-based immunotherapy against cancer [23]. Generally, one to several million DC are used per single injection, and this procedure is repeated in defined intervals for several times [24]. Good manufacturing practice (GMP) guidelines demand regular quality controls of DC before administration, resulting in higher DC cell demand during the manufacturing process. In several clinical trials, even cautiously calculated requirements for DC cell numbers were not achieved [25, 26], or all available DC per patient were administered resulting in a high variability of injected cell numbers [27–29]. Therefore, cryopreservation of PBMC by CRF aims to ensure an improved DC yield as one central prerequisite for successful DC immunotherapy.

Plastic adherence of PBMC obtained from leukapheresis for generation of monocyte-derived DC is the most widely used technique because of easy handling and cost-effectiveness and has been extensively validated and optimized for closed cell culture systems according to GMP guidelines for patient application [30, 31]. However, the variability in DC purity after this approach was addressed by recent publications [32–34], and other technologies such as positive and negative immunomagnetic selection and monocyte elutriation, on the basis of counter-flow centrifugation, have been evaluated as alternatives for cell preparation [33, 35]. Nevertheless, since methods differ significantly in need of financial and human resources, and concerns exist regarding activation signals by extended pretreatment or labeling with xenogeneic antibodies [36, 37], the optimal isolation method for DC generation it is a matter of ongoing controversy [3]. During the years 1997–2008, more than 600 melanoma patients have been treated with DC immunotherapy, the vast majority of patients received monocyte-derived DC after plastic-adherence of PBMC [24]. For evaluation of more recent trials, our literature research retrieved 32 published immunotherapy trials using dendritic cells (MEDLINE, search terms: dendritic cells, immunotherapy, limits: clinical trials, English language, date range: 01/01/2008-01/01/2010). Three clinical trials out of these were not accessible for the detailed study protocol. The method of plastic adherence for monocyte isolation was used in 23/29 trials, magnetic beads for cell isolation in 5/29 studies, and elutriation in one trial. Remarkably, all researchers working with cryopreserved cells did not use controlled-rate freezing procedures.

We developed further the results of one earlier study that showed the superiority of cryopreserved monocytes over pre-differentiated iDC or mDC for DC immunotherapy [12]. In our experiments, we continued to increase DC yields by introducing a computer-assisted controlled-rate freezer (CRF). Our finding that total protein per plate of IPA-PBMC is already significantly reduced 24 h after culture of adherent cells led to the hypothesis that fewer IPA-PBMC recover compared with CRF-PBMC. It was shown that viability of cells after standard freezing procedures was significantly reduced [38]. Another contributing factor for reduced IPA-iDC numbers might be cryopreservation-dependent downregulation of adherence molecules on monocytes and therefore an increased loss of CD14+ cells during pre-plating. Earlier studies suggested the involvement of CD62L (L-selectin), as it mediates monocyte adhesion to cytokine-stimulated endothelial monolayers [39], and its downregulation was regularly observed during cryopreservation of hematopoietic stem cells and PBMC [40, 41].

The applied method of CRF for cryopreservation of PBMC was analyzed in one earlier study. The authors concluded that CRF-PBMC can be stored for at least 12 years with no general tendency toward cell loss and no statistically significant changes in the percentage of viable cells in correlation with the time of storage [42]. Interestingly, the authors also observed that IPA-PBMC had an estimated loss of 2.23 % of viability per year, compared with a significant lower 0.01 % reduction of viability per year for CRF-PBMC. Therefore, the selected method of cryopreservation does not only affect the cells at the time of the procedure but shows a prolonged influence on quantity and quality of cryopreserved cells depending on the time of storage.

Our comparative phenotypical analysis of NC-iDC, IPA-iDC, and CRF-iDC and corresponding mDC did not reveal significant differences in the expression of surface molecules including CD83 and CD14. As expected, the induction of maturation by poly(I/C) resulted in statistically significant differences in CD83 expression between immature and mature DC [43]. Therefore, our results confirm an earlier study that also reported no phenotypical differences between NC-DC and DC generated from CRF-PBMC [38].

In addition, after the different methods of cryopreservation, we found no differences in cytokine secretion during DC maturation by semi-quantitative cytokine array screening for 36 cytokines with two exceptions. First, IL12p70 secretion into the supernatant was lower for NC-DC and higher for CRF-DC. However, exact quantification by ELISA technique did not show significance of this observation. To the best of our knowledge, no other studies comparing IL12p70 secretion of differently cryopreserved DC after maturation by poly(I/C) have been published. Second, CXCL1 (Groα) secretion was twofold higher by NC-DC compared with cryopreserved DC on array analysis. This finding was not further evaluated in our experiments because of the rather limited role of CXCL1 in DC chemotaxis [44].

Phenotypical and functional analysis of DC after the different cryopreservation protocols was completed by assays of allogeneic and autologous T-cell stimulation. Proliferation assays of IPA-DC and CRF-DC with allogeneic CFDA-SE labeled T cells revealed no differences in stimulatory capacity. Remarkably, after 1 week of re-stimulation of autologous PBMC with antigen-loaded mDC for expansion of specific T cells, determination of specific IFNγ-spots resulted in significantly more spots for CRF-mDC than for IPA-mDC. Moreover, three of six IPA-mDC batches (in two of three donors) and none of six CRF-mDC batches failed to induce an autologous T-cell response with antigen-specific IFNγ-spots. Given that the induction of specific autologous T-cell responses is crucial in DC-based immunotherapy [4], this observation might be important for the design of future clinical studies. Although several studies emphasized that cryopreservation does not alter phenotypic or functional properties of DC [9, 45, 46], these studies evaluated already differentiated iDC or mDC for IPA-cryopreservation and may not reflect all aspects of cryopreserving cells for DC immunotherapy.

In conclusion, this study demonstrates that automated controlled-rate freezing of PBMC for DC generation results in higher cell yields of immature DC as compared with standard uncontrolled freezing of cells. Moreover, CRF-DC do not differ from DC after standard uncontrolled freezing or freshly prepared DC regarding the expression of relevant surface markers, the potential of full DC maturation, cytokine production, and allogeneic T-cell stimulatory capacity. Importantly, DC generated from CRF-cryopreserved PBMC induced a significantly higher antigen-specific IFN-γ release from autologous effector T cells in ELISPOT assays as compared with DC after standard freezing procedures.

## References

[1] Steinman RM, Banchereau J (2007). Taking dendritic cells into medicine. Nature.

[2] Trombetta ES, Mellman I (2005). Cell biology of antigen processing in vitro and in vivo. Annu Rev Immunol.

[3] Palucka K, Ueno H, Banchereau J (2011). Recent developments in cancer vaccines. J Immunol.

[4] Finn OJ (2008). Cancer immunology. N Engl J Med.

[5] Kantoff PW, Higano CS, Shore ND, Berger ER, Small EJ, Penson DF (2010). Sipuleucel-T immunotherapy for castration-resistant prostate cancer. N Engl J Med.

[6] Boscardin SB, Hafalla JC, Masilamani RF, Kamphorst AO, Zebroski HA, Rai U (2006). Antigen targeting to dendritic cells elicits long-lived T cell help for antibody responses. J Exp Med.

[7] Thomson AW, Robbins PD (2008). Tolerogenic dendritic cells for autoimmune disease and transplantation. Ann Rheum Dis.

[8] Rinaldo CR (2009). Dendritic cell-based human immunodeficiency virus vaccine. J Intern Med.

[9] Feuerstein B, Berger TG, Maczek C, Roder C, Schreiner D, Hirsch U (2000). A method for the production of cryopreserved aliquots of antigen-preloaded, mature dendritic cells ready for clinical use. J Immunol Methods.

[10] Garritsen HS, Macke L, Meyring W, Hannig H, Pagelow U, Wormann B (2010). Efficient generation of clinical-grade genetically modified dendritic cells for presentation of multiple tumor-associated proteins. Transfusion.

[11] John J, Dalgleish A, Melcher A, Pandha H (2005). Cryopreserved dendritic cells for intratumoral immunotherapy do not require re-culture prior to human vaccination. J Immunol Methods.

[12] Hayden H, Friedl J, Dettke M, Sachet M, Hassler M, Dubsky P (2009). Cryopreservation of monocytes is superior to cryopreservation of immature or semi-mature dendritic cells for dendritic cell-based immunotherapy. J Immunother.

[13] Meryman HT (1956). Mechanics of freezing in living cells and tissues. Science.

[14] Pegg DE (2002). The history and principles of cryopreservation. Semin Reprod Med.

[15] Meryman HT (2007). Cryopreservation of living cells: principles and practice. Transfusion.

[16] Perez-Oteyza J, Bornstein R, Corral M, Hermosa V, Alegre A, Torrabadella M (1998). Controlled-rate versus uncontrolled-rate cryopreservation of peripheral blood progenitor cells: a prospective multicenter study. Group for cryobiology and biology of bone marrow transplantation (CBTMO), Spain. Haematologica.

[17] Montanari M, Capelli D, Poloni A, Massidda D, Brunori M, Spitaleri L (2003). Long-term hematologic reconstitution after autologous peripheral blood progenitor cell transplantation: a comparison between controlled-rate freezing and uncontrolled-rate freezing at 80 °C. Transfusion.

[18] Bakken AM (2006). Cryopreserving human peripheral blood progenitor cells. Curr Stem Cell Res Ther.

[19] Wolf CE, Meyer M, Riggert J (2005). Leukapheresis for the extraction of monocytes and various lymphocyte subpopulations from peripheral blood: product quality and prediction of the yield using different harvest procedures. Vox Sang.

[20] Haenssle H, Buhl T, Knudsen S, Krueger U, Rosenberger A, Reich K (2008). CD40 ligation during dendritic cell maturation reduces cell death and prevents interleukin-10-induced regression to macrophage-like monocytes. Exp Dermatol.

[21] Brunner E, Domhof S, Langer F (2002). Nonparametric analysis of longitudinal data in factorial experiments.

[22] Verdijk P, Aarntzen EH, Lesterhuis WJ, Boullart AC, Kok E, van Rossum MM (2009). Limited amounts of dendritic cells migrate into the T-cell area of lymph nodes but have high immune activating potential in melanoma patients. Clin Cancer Res.

[23] Draube A, Klein-Gonzalez N, Mattheus S, Brillant C, Hellmich M, Engert A (2011). Dendritic cell based tumor vaccination in prostate and renal cell cancer: a systematic review and meta-analysis. PLoS ONE.

[24] Engell-Noerregaard L, Hansen TH, Andersen MH, Thor Straten P, Svane IM (2009). Review of clinical studies on dendritic cell-based vaccination of patients with malignant melanoma: assessment of correlation between clinical response and vaccine parameters. Cancer Immunol Immunother.

[25] Lesterhuis WJ, De Vries IJ, Schreibelt G, Schuurhuis DH, Aarntzen EH, De Boer A (2010). Immunogenicity of dendritic cells pulsed with CEA peptide or transfected with CEA mRNA for vaccination of colorectal cancer patients. Anticancer Res.

[26] Schadendorf D, Ugurel S, Schuler-Thurner B, Nestle FO, Enk A, Brocker EB (2006). Dacarbazine (DTIC) versus vaccination with autologous peptide-pulsed dendritic cells (DC) in first-line treatment of patients with metastatic melanoma: a randomized phase III trial of the DC study group of the DeCOG. Ann Oncol.

[27] Ribas A, Comin-Anduix B, Chmielowski B, Jalil J, de la Rocha P, McCannel TA (2009). Dendritic cell vaccination combined with CTLA4 blockade in patients with metastatic melanoma. Clin Cancer Res.

[28] Rollig C, Schmidt C, Bornhauser M, Ehninger G, Schmitz M, Auffermann-Gretzinger S (2011). Induction of cellular immune responses in patients with stage-I multiple myeloma after vaccination with autologous idiotype-pulsed dendritic cells. J Immunother.

[29] Yi Q, Szmania S, Freeman J, Qian J, Rosen NA, Viswamitra S (2010). Optimizing dendritic cell-based immunotherapy in multiple myeloma: intranodal injections of idiotype-pulsed CD40 ligand-matured vaccines led to induction of type-1 and cytotoxic T-cell immune responses in patients. Br J Haematol.

[30] Tuyaerts S, Aerts JL, Corthals J, Neyns B, Heirman C, Breckpot K (2007). Current approaches in dendritic cell generation and future implications for cancer immunotherapy. Cancer Immunol Immunother.

[31] Vuckovic S, Clark GJ, Hart DN (2002). Growth factors, cytokines and dendritic cell development. Curr Pharm Des.

[32] Suen Y, Lee SM, Aono F, Hou S, Loudovaris M, Ofstein G (2001). Comparison of monocyte enrichment by immuno-magnetic depletion or adherence for the clinical-scale generation of DC. Cytotherapy.

[33] Felzmann T, Witt V, Wimmer D, Ressmann G, Wagner D, Paul P (2003). Monocyte enrichment from leukapheresis products for the generation of DCs by plastic adherence, or by positive or negative selection. Cytotherapy.

[34] Meyer-Wentrup F, Burdach S (2003). Efficacy of dendritic cell generation for clinical use: recovery and purity of monocytes and mature dendritic cells after immunomagnetic sorting or adherence selection of CD14+ starting populations. J Hematother Stem Cell Res.

[35] Perseghin P, D’Amico G, Dander E, Gaipa G, Dassi M, Biagi E (2008). Isolation of monocytes from leukapheretic products for large-scale GMP-grade generation of cytomegalovirus-specific T-cell lines by means of an automated elutriation device. Transfusion.

[36] Breckpot K, Corthals J, Heirman C, Bonehill A, Michiels A, Tuyaerts S (2004). Activation of monocytes via the CD14 receptor leads to the enhanced lentiviral transduction of immature dendritic cells. Hum Gene Ther.

[37] Elkord E, Williams PE, Kynaston H, Rowbottom AW (2005). Human monocyte isolation methods influence cytokine production from in vitro generated dendritic cells. Immunology.

[38] Heo YJ, Son CH, Chung JS, Park YS, Son JH (2009). The cryopreservation of high concentrated PBMC for dendritic cell (DC)-based cancer immunotherapy. Cryobiology.

[39] Spertini O, Luscinskas FW, Gimbrone MA, Tedder TF (1992). Monocyte attachment to activated human vascular endothelium in vitro is mediated by leukocyte adhesion molecule-1 (L-selectin) under nonstatic conditions. J Exp Med.

[40] De Boer F, Drager AM, Van der Wall E, Pinedo HM, Schuurhuis GJ (1998). Changes in L-selectin expression on CD34-positive cells upon cryopreservation of peripheral blood stem cell transplants. Bone Marrow Transpl.

[41] Weinberg A, Song LY, Wilkening C, Sevin A, Blais B, Louzao R (2009). Optimization and limitations of use of cryopreserved peripheral blood mononuclear cells for functional and phenotypic T-cell characterization. Clin Vaccine Immunol.

[42] Kleeberger CA, Lyles RH, Margolick JB, Rinaldo CR, Phair JP, Giorgi JV (1999). Viability and recovery of peripheral blood mononuclear cells cryopreserved for up to 12 years in a multicenter study. Clin Diagn Lab Immunol.

[43] Haenssle HA, Riedl P, Buhl T, Schardt A, Rosenberger A, Schön MP (2010). Intracellular delivery of major histocompatibility complex class I-binding epitopes: dendritic cells loaded and matured with cationic peptide/poly(I:C) complexes efficiently activate T cells. Exp Dermatol.

[44] Alvarez D, Vollmann EH, von Andrian UH (2008). Mechanisms and consequences of dendritic cell migration. Immunity.

[45] Westermann J, Korner IJ, Kopp J, Kurz S, Zenke M, Dorken B (2003). Cryopreservation of mature monocyte-derived human dendritic cells for vaccination: influence on phenotype and functional properties. Cancer Immunol Immunother.

[46] Hori S, Heike Y, Takei M, Maruyama M, Inoue Y, Lee JJ (2004). Freeze-thawing procedures have no influence on the phenotypic and functional development of dendritic cells generated from peripheral blood CD14+ monocytes. J Immunother.

